# Meiotic chiasmata variations in the H genome among Triticeae species of varying ploidy

**DOI:** 10.3389/fpls.2025.1666216

**Published:** 2025-10-15

**Authors:** Ying Yang, Bo Liu, Jie Chen, Jialei Guo, Wenjie Shen, Yixin Meng, Quanwen Dou

**Affiliations:** ^1^ Key Laboratory of Adaptation and Evolution of Plateau Biota, Northwest Institute of Plateau Biology, Chinese Academy of Sciences, Xining, Qinghai, China; ^2^ University of Chinese Academy of Sciences, Beijing, China; ^3^ Qinghai Provincial Key Laboratory of Crop Molecular Breeding, Northwest Institute of Plateau Biology, Chinese Academy of Sciences, Xining, Qinghai, China

**Keywords:** polyploid, autopolyploid, allopolyploid, chiasmata, *Hordeum*

## Abstract

Meiotic chiasmata are critical for genetic diversity and chromosome segregation. This study aimed to cytologically analyze the variations in chiasmata within the H genome across different ploidy levels, specifically in diploid (*Hordeum bogdanii*), autotetraploid (*Hordeum brevisubulatum*), and allotetraploid (*Elymus sibiricus*) species, to understand the impact of polyploidization. We conducted a comparative cytological analysis of meiotic chiasmata in the H genome of the three species during diakinesis and metaphase I. This study revealed significant variations in the types and frequencies of chromosomal pairing configurations both across different species and among chromosomes within the same species. *H. brevisubulatum* exhibited a high frequency of quadrivalents. The number of chiasmata in the H genome decreased from 21.32 in *H. bogdanii* to 19.00 in *E. sibiricus* and 14.67 in *H. brevisubulatum* per genome during diakinesis, with a further significant reduction observed at metaphase I. All chromosomes exhibited a similar reduction in chiasmata number from diploid to tetraploid, with the exception of chromosome 1H, which showed a significant increase in *E. sibiricus* during diakinesis. The frequency of chiasmata significantly decreased from *H. bogdanii* to *E. sibiricus* and *H. brevisubulatum* in both the terminal and interstitial regions. Chiasmata in *E. sibiricus* and *H. brevisubulatum* were more terminally localized compared to those in *H. bogdanii*. However, a significant increase in chiasmata frequency was observed on the short arms of chromosomes 1H and 4H in *E. sibiricus* during diakinesis. Various patterns of chiasmata localization were observed across the three species during diakinesis. In *E. sibiricus*, interstitial chiasmata were distributed more distally along both chromosomal arms compared to those in *H. bogdanii*. In contrast, interstitial chiasmata were absent on the short arms of most chromosomes in *H. brevisubulatum* and exhibited a more proximal distribution on the long arms. The evolutionary and adaptive significance of these chiasmata variations during polyploidization was further discussed.

## 1 Introduction

Meiosis is the primary process in the sexual life cycle of most eukaryotes, during which haploid gametes are generated from diploid germ cells. It involves one round of DNA replication, followed by two rounds of chromosome segregation. Cytological homologous chromosome pairing is a crucial step in meiosis I, involves key events, such as chromosomal recombination and crossover interference. At least one chiasma (obligatory chiasma) per bivalent is required to ensure proper chromosomal segregation, while multiple chiasmata are associated with a high frequency of genetic recombination at this stage (Jones and Franklin, 2006; Zickler and Kleckner, 2023). During meiosis, crossover rates (recombination) are unevenly distributed along chromosomes, thereby influencing the generation of novel genotypes and the efficacy of selection (Brazier et al., 2022). Owing to the positive association between recombination and gene density, crossover patterns are considered critical for efficient chromosomal shuffling, however, the evolutionary significance of diverse recombination patterns remains unclear (Brazier and Glémin, 2022).

Polyploidy, or whole-genome duplication (WGD), comprises two primary forms, allopolyploidy, which arises from interspecific hybridization and genome doubling, and autopolyploidy, which results from genome doubling within a species. Both mechanisms play a significant role in plant evolution (Soltis et al., 2015; Heslop-Harrison et al., 2023). Meiosis in polyploids is challenging owing to the presence of more than two homologs. Cytological diploidization of autopolyploids can be accomplished by crossover frequency reduction and redistribution (Grandont et al., 2013; Yant et al., 2013; Bomblies et al., 2015). In most established allopolyploids, bivalents form predominantly within each subgenome, and disomic inheritance is maintained due to persistent heterozygosity between subgenomes (Pikaard, 2001; Bomblies and Madlung, 2014). However, neo-allopolyploids exhibit extensive homeologous recombination (Zhang et al., 2013). Additionally, newly formed autopolyploids and allopolyploids exhibit an increased number of crossover events (Pecinka Alex et al., 2011). Polyploidy may promote meiotic recombination, and the consequent rapid generation of genetic diversity likely contributes to its prevalence (Pecinka Alex et al., 2011). High recombination rates are associated with recombination hotspots and gene-rich and diverse regions (Lawrence et al., 2017; Brazier and Glémin, 2022). Variations in the crossover rate and redistribution may significantly affect the shifting of genetic diversity, such as recombination hot spot, although they may enhance the adaptive potential of closely related species in challenging natural environments (Smukowski and Noor, 2011). Evaluating meiotic recombination in species with varying ploidy levels from a stable and long-term-adapted taxon could help clarify how recombination variation during polyploidization contributed to the enhanced adaptability of polyploids.

Triticeae (Poaceae) contains approximately 100 annual and 400 perennial taxa (Dewey, 1983). This economically crucial tribe includes genera, such as *Triticum*, *Hordeum*, and *Secale*, which include widely domesticated crops like wheat, barley, and rye. This tribe includes species with varying ploidy levels, serving as both a vital genetic resource for food security and a prominent model system for investigating the interactions among hybridization, chromosomal evolution, and biological diversification (Parisod and Badaeva, 2020; Kroupin et al., 2023). *Hordeum bogdanii* is a diploid species (2n = 2x = 14) with an H genome, whereas *H. brevisubulatum* is an autopolyploid (2n = 28) (Landström et al., 1984; Al-Saghir, 2016). The similar cytological patterns between the two species indicate that *H. brevisubulatum* originated as an autopolyploid from a *Hordeum* species carrying the common H genome, such as *H. bogdanii* (Dou et al., 2016). *Elymus sibiricus L.* is an allopolyploid with a genome constitution of StStHH (2n = 28), where St and H are derived from *Pseudoroegneria* (Neveski) Löve and *Hordeum* L., respectively (Dewey, 1983). *H. bogdanii* is a widely distributed Asiatic species, ranging from western Iran to eastern China (Yang et al., 1987), whereas *H. brevisubulatum* is distributed from Western Turkey to eastern China (Bothmer, 1979). Both species show superior adaptability to saline-alkaline soils (Garthwaite et al., 2005; Zhang et al., 2020). *E. sibiricus* is widely distributed across the Northern Hemisphere, with particular preponderance in Sweden, northern Asia, Japan, and North America (Baum et al., 2012). It usually grows on moist meadows, riparian sands, among open woodland, on sunny or semi-shade mountains or valley slopes at elevations ranging from 1,000 to 4,000 meters (Lu, 1993). The superior adaptability of the H genome to saline-alkaline soils is not distinctly manifested in *E. sibiricus*. Investigation on the crossover variation among three closely related Triticeae species, which share a common progenitor H genome, may provide valuable insights into their diverse adaptive strategies. Due to the large chromosome size in the Triticeae species, chiasmata resulting from crossing over are clearly visible during diakinesis, with each chiasma representing a single crossover (Fu and Sears, 1973), chromosome synteny across species can be clearly determined cytologically (Danilova et al., 2012; Liu et al., 2023). In this study, the variation in chiasmata of the H genome was analyzed cytologically during diakinesis and metaphase I across species of different ploidy levels. Chromosomal synteny of the H genome in was ambiguously determined in *H. hordeum* (2H), *H. brevisubulatum*, and *E, sibiricus*. The results of the variations in the crossover rate and redistribution at the chromosomal level provide important insights into the evolution and adaptation of the H genome during polyploidization, facilitating further genomic and molecular investigations.

## 2 Materials and methods

### 2.1 Plant materials

Three plant species—*Hordeum brevisubulatum* (Trin.) Link, *H. bogdanii* Wilensky, and *Elymus sibiricus* L.—were used in this study. The accessions of *H. bogdanii* and *E. sibiricus*, collected from a wetland in Golmud and an arid grassland in Tongde, Qinghai, China, respectively, were similar to those used in a previous study (Liu et al., 2023). *H. brevisubulatum* accessions were collected from the same region as *E. sibiricus*. All plants were grown in a common garden in Xining, Qinghai Province, China.

### 2.2 Meiotic chromosome preparation

Pollen mother cell (PMC) collection and chromosome preparation were performed as described by Liu et al. (2021) for *Elymus nutans*. Inflorescences were collected at the early flowering stage, characterized morphologically by a distance of 1–2 cm between the flag leaf and the subsequent leaf. The collected samples were fixed in Carnoy’s solution II (ethanol: glacial acetic acid: chloroform = 6:1:3) for 24 hours and then stored in 70% ethanol at -20 °C until further use. Initially, pollen mother cells (PMCs) were squashed in 45% acetic acid and examined under a phase-contrast microscope to identify meiotic stages. Slides containing ideal target cells were labeled and stored at -80 °C for subsequent analysis.

### 2.3 FISH probes

Fourteen cDNA sequences, previously mapped to the distal ends of each arm of the seven homoeologous chromosomes in wheat by fluorescence *in sit* hybridization (FISH) (Danilova et al., 2014) and later confirmed in *Hordeum* and *Elymus* species Liu et al. (2023), were selected for this study. These sequences were obtained from the Triticeae Full-length cDNA (FlcDNA) database (http://www.shigen.nig.ac.jp/wheat/komugi/ests/tissueBrowse.jsp) and kindly provided by the National BioResource Project-Wheat, Japan (https://nbrp.jp/en/) (**Supplementary Table S1**). The cDNA probes were labeled according to the procedures described by Liu et al. (2023).

Synthetic oligonucleotides representing 5S rDNA, 45S rDNA (Nomar Espinosa et al., 2018), pAs1, and a microsatellite (AAG)_10_ (Tang et al., 2014) (**Supplementary Table S2**) were end-labeled with fluorescein amidite (green) or carboxy tetramethyl rhodamine (red) (Sangon Biotech Co., Ltd., Shanghai, China).

### 2.4 FISH

Slide denaturation, preparation of the hybridization mixture, and hybridization were performed according to Liu et al. (2022). The sequential FISH procedure was conducted as follows: Following imaging of the first set of probes, the cover slips were removed, and the slides were washed in 2× SSC at room temperature for 20 min and briefly dried. The hybridization mixture for the second round of FISH was then applied directly to the slides without an additional denaturation step. All subsequent procedures were identical to those of the first hybridization. Images were captured using a cooled charge-coupled device camera (DP80) under a fluorescence microscope (Olympus BX63). Finally, the images were adjusted using Adobe Photoshop CC 2015 (https://www.adobe.com) for contrast and background optimization.

### 2.5 Statistical analysis

First, the pairing configurations of different homologous chromosomes were determined in each cell during meiotic diakinesis or metaphase I. Second, the number of chiasmata in different homologous chromosomal pairs was counted in each cell. Over 21 PMCs were captured at the targeted meiotic stage for each accession. The frequencies of different pairing configurations and chiasmata numbers for specific homologous chromosomes were summarized and averaged across all the cells. Because *H. brevisubulatum* is an autoploid species with two pairs of homologous chromosomes, the numerical values were divided by two to normalize them against the single pair of homologous chromosomes in *H. bogdanii* and *E. sibiricus*. One-way analysis of variance was performed to compare the significant differences in chiasmata across species or chromosomes using OriginPro 2019 (https://www.originlab.com/2019). Graphs illustrating chiasma numbers were generated using GraphPad Prism 8 (https://www.graphpad-prism.cn/).

## 3 Results

### 3.1 Identification of homeologous chromosomes of the H genome across different species

**Figure 1:**
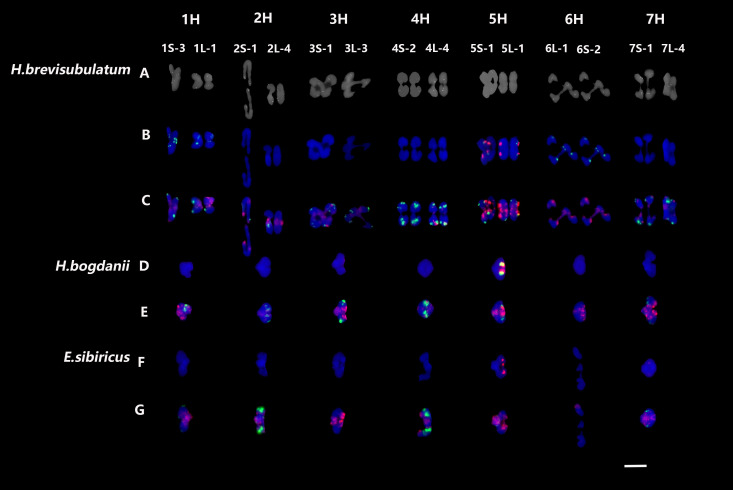
FISH patterns of individual meiosis chromosomes during or metaphase I in three species. **(A)** Probed with single-gene probes; **(B, D, F)** probed with 45S rDNA (green) and 5S rDNA (red); **(C, E, G)** probed with pAs1 (red) and (AAG)_10_ (green). Bar = 10 μm.

The homoeologous chromosomes of *H. bogdanii* and *E. sibiricus* were well characterized using cDNA and repetitive sequence probes (Liu et al., 2023). Because homoeologous chromosome data of *H. brevisubulatum* are lacking, chromosome identification was performed using cDNA and other repetitive sequences, such as 45S rDNA, 5S rDNA, pAs1, and (AAG)_10_ probes (**Supplementary Figure S1**). Mapping of 14 cDNA probes—each representing one arm of the seven chromosomes—demonstrated that the collinearity of the chromosomes of *H. brevisubulatum* was highly conserved. Thus, in the present study, homoeologous chromosomes during meiotic diakinesis or metaphase I in three species were unambiguously identified using a combination of probes for 45S rDNA, 5S rDNA, pAs1, and (AAG)_10_ using sequential FISH (**Supplementary Figure S2**). Therefore, the homoeologous chromosomes of the H genome during meiotic diakinesis or metaphase I were well characterized across the three species using four repetitive sequence probes (**Figure 1**).

### 3.2 Variations in chromosome pairing configuration within and across species

**Figure 2:**
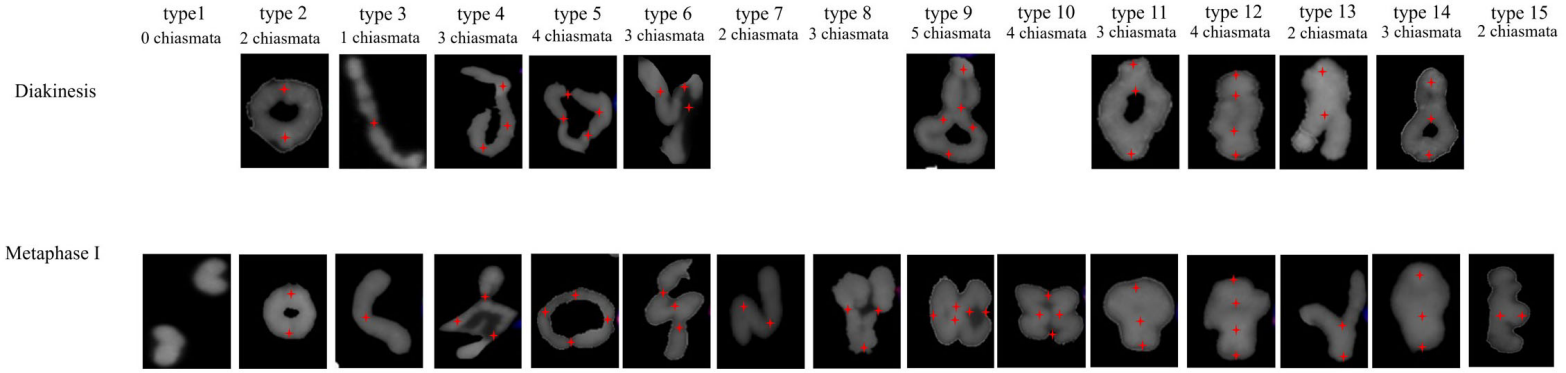
Chromosome pairing configuration types during diakinesis and metaphase I. 1. Univalent; 2. Bivalent terminal rings; 3. Bivalent terminal rod; 4. Quadrivalent terminal chain; 5. Quadrivalent terminal ring; 6. N-shaped quadrivalent; 7. N-shaped trivalent; 8. V-shaped quadrivalent; 9. 8-shaped quadrivalent; 10. 8&3 shaped quadrivalents; 11. Bivalent proximal terminal ring; 12. Bivalent pericentric rings; 13. Y-shaped bivalent; 14. 8-shaped bivalent; and 15. Bivalent proximal terminal rod. Red crosses (+) indicate chiasmata.

Analysis of the chromosomal pairing configurations across different cells revealed variability within the species and between species during both diakinesis and metaphase I. Fifteen pairing configurations were classified based on the total number of cells examined (**Figure 2**). Among these, 10 configuration types were common to both meiotic stages, whereas 5 types were exclusively observed during metaphase I. Configuration type 1 was univalent without chiasmata; types 2 and 3 were bivalent with chiasmata at the terminal regions; types 4, 5, 6, 7, 8, 9, and 10 were quadrivalents or trivalent; and types 11, 12, 13, 14, and 15 were bivalent with both terminal and proximal chiasmata (**Figure 2**).

A comparison of the configuration type frequencies per cell demonstrated that during the diakinesis stage, *H. bogdanii* exhibited the highest frequency for type 11 (51.88%), followed by type 12 (24.06%) among 38 meiocytes examined. *H. brevisubulatum* showed the highest frequency of type 2 (54.30%), followed by type 9 (27.27%), across 36 investigated meiocytes. *E. sibiricus* exhibited the highest frequency for type 14 (40.82%), followed by type 2 (31.29%), across 21 investigated meiocytes (**Supplementary Table S3**). This indicates that the chromosomal pairing configurations of *H. bogdanii* are characterized by high frequencies of bivalents with both terminal and proximal chiasmata, whereas those in *H. brevisubulatum* are predominantly quadrivalents. In metaphase I, the dominant configuration types shifted. Type 2 was a common dominated configuration across all three species. However, type 11 was more prevalent in *H. bogdanii* than type 2, and types 9 and 11 were less common in *H. brevisubulatum* and *E. sibiricus* respectively than type 2 (**Supplementary Tables S3, S4**). Additionally, variations in configuration type frequency were observed between different chromosomes within and across species (**Supplementary Tables S3, S4**) at both stages. For example, during diakinesis, the chromosome 1H of *H. bogdanii* exhibited the highest frequency for types 11 and 2 at 44.74%. In contrast, chromosome 3H exhibited a predominance of type 11 (78.95%), while type 2 observed at a frequency of 2.63%.

### 3.3 Chiasmata number variation across species

**Figure 3:**
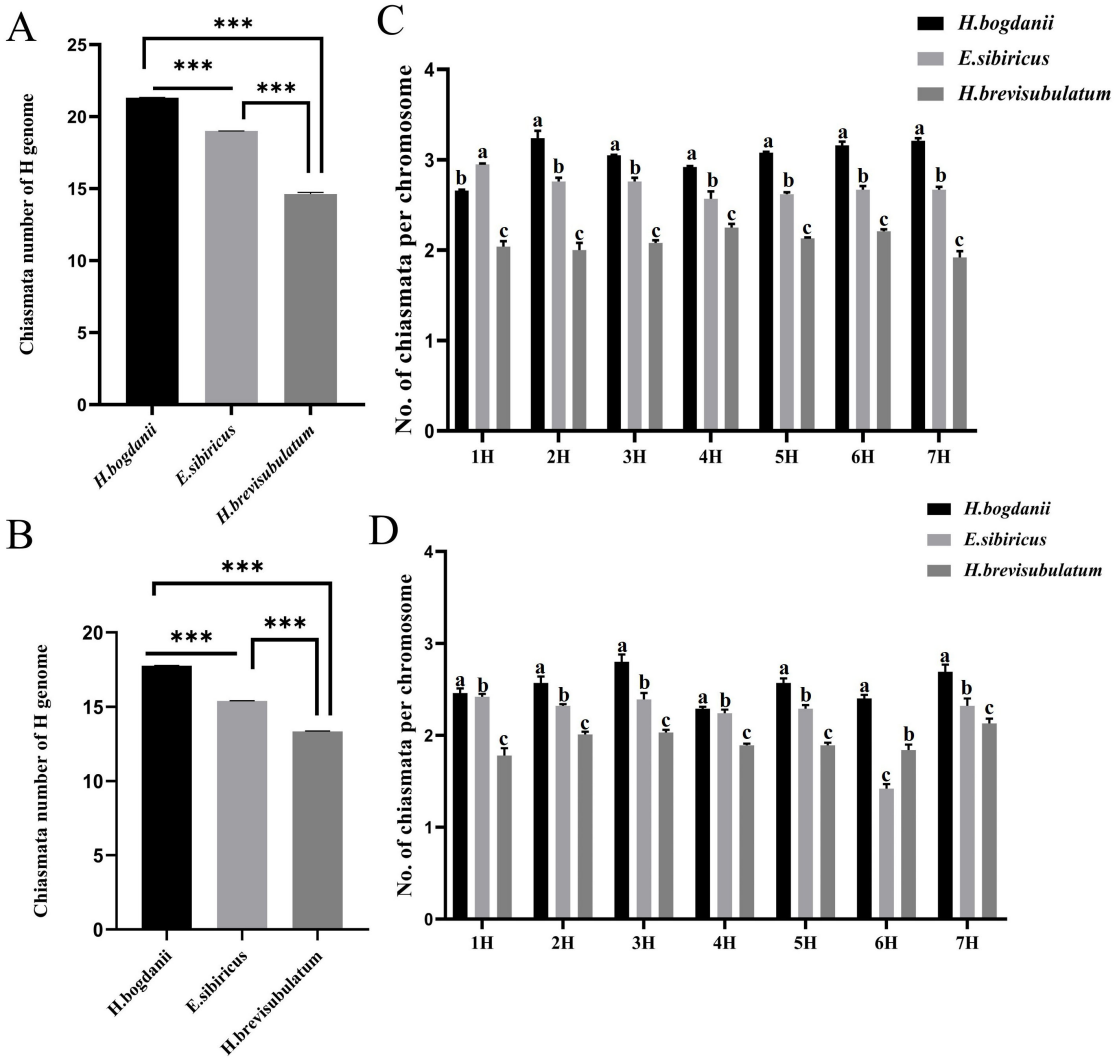
Analysis of variance of chromosome chiasmata number during meiotic diakineses and metaphaes I. **(A)** Total number of chiasmata number in the H genome between species at diakinesis; **(B)** Total number of chiasmata in the total H genome between species at metaphaes I **(C)** Number of chiasmata on same homoeologous chromosomes between different species at diakineses. **(D)** Number of chiasmata on same homoeologous chromosomes between species at metaphaes I. Species involved: *H. bogdanii*, *E. sibiricus*, and *H. brevisubulatum*. Asterisks in A indicate significant differences (*p* < 0.001), whereas different Roman letters in each column of B indicate significant differences between two means (*p* < 0.05).

Statistical analysis of the H genomes during diakinesis revealed the following chiasmata number per genome: *H. bogdanii* (21.32), *E. sibiricus* (19.00), and *H. brevisubulatum* (14.67) (**Figure 3A**). This corresponds to 3.05, 2.71, and 2.10 chiasmata per chromosome pair in *H. bogdanii, E. sibiricus*, and *H. brevisubulatum*, respectively. During diakinesis, the number of chiasmata decreased significantly from diploid to tetraploid species, with a significant reduction observed in autopolyploids. The number of chiasmata at metaphase I was significantly reduced compared to diakinesis due to chiasma terminalization. The variations in chiasmata number during metaphase I followed a trend similar to that observed during diakinesis, with significant differences among the species: *H. bogdanii* (17.78 ± 0.06 per cell) > *E. sibiricus* (15.40 ± 0.05 per cell) > *H. brevisubulatum* (13.57± 0.04 per cell) (**Figure 3B**).

As each H genome chromosome in the three species was clearly recognized, variations in the chiasmata number were further compared for each homoeologous chromosome across different species. In the diakinesis stage, the number of chiasmata in all chromosomes of *H. bogdanii* was significantly higher than that in the corresponding homoeologous chromosomes of *E. sibiricus* and *H*. *brevisubulatum*, except for chromosome 1H in *E. sibiricus*, which exhibited a significantly higher chiasmata number than that in *H. bogdanii*. Similarly, the chiasmata number in all chromosomes in *E. sibiricus* was significantly higher than that in *H*. *brevisubulatum* (**Figure 3C**). In metaphase I, the chiasmata number of all homeologous chromosomes differed significantly in the following order: *H. bogdanii > E. sibiricus* > *H*. *brevisubulatum*, except that the chromosome 6H of *E. sibiricus* showed a lower number compared to both *H. bogdanii* and *H*. *brevisubulatum* (**Figure 3D**).

### 3.4 Chiasmata localization and occurrence of frequency variation

Chiasmata distribution patterns were broadly categorized into terminal and interstitial sites in each chromosomal arm to distinguish pairing configurations and the enumeration of chiasmata. However, different chromosomes were recognized more accurately using repetitive sequences as land markers. The chiasmata locations were more accurately determined using cytological markers (pAs1 and [AAG]_10_) and were further quantified by calculating the ratio of the distance from the end of the short or long arm to the chiasma location divided by the total chromosome length. Additionally, the frequencies of localized chiasmata were calculated as the ratio of their occurrence on specific chromosomes to the total number of cells assessed. The ratio of the frequency of terminal to interstitial chiasmata (FT/FI) was used to determine the chiasmata distribution patterns (**Tables 1**, **2**).

**Table 1:** Chiasmata locations and occurrence frequencies (%) at the diakinesis in three species.

Species	Chromosome	Diakinesis							
		No chiamata	Terminal chiasmata			Interstitial chiasmata			Ratio (FT/FI)
			Short-arm	Long-arm	Average	Short-arm	Long-arm	Average	
*H. bogdanii*	1H	0.00	100.00	100.00	100.00	34.00(37.33)	32.00(35.61)	33.00	3.03
	2H	0.00	97.00	100.00	98.50	39.00(36.83)	87.00(40.44)	63.00	1.56
	3H	0.00	95.00	100.00	97.50	13.00(34.94)	97.00(35.01)	55.00	1.77
	4H	0.00	100.00	100.00	100.00	34.00(29.65)	58.00(34.04)	46.00	2.17
	5H	0.00	100.00	100.00	100.00	47.00(34.31)	61.00(40.41)	54.00	1.85
	6H	0.00	100.00	100.00	100.00	45.00(39.92)	71.00(33.07)	58.00	1.72
	7H	0.00	100.00	100.00	100.00	37.00(33.63)	84.00(35.14)	60.50	1.65
	Average	0.00	98.86	100.00	99.43	35.57(35.23)	70.00(36.25)	52.79	1.88
*E. sibiricus*	1H	0.00	100.00	100.00	100.00	71.00(35.62)	24.00(36.32)	47.50	2.11
	2H	0.00	90.00	95.00	92.50	19.00(30.34)	71.00(28.98)	45.00	2.06
	3H	0.00	100.00	100.00	100.00	5.00(33.29)	71.00(32.25)	38.00	2.63
	4H	0.00	90.00	100.00	95.00	52.00(34.27)	14.00(36.16)	33.00	2.88
	5H	0.00	95.00	100.00	97.50	5.00(35.62)	62.00(40.56)	33.50	2.91
	6H	0.00	95.00	95.00	95.00	10.00(34.42)	67.00(35.47)	38.50	2.47
	7H	0.00	100.00	100.00	100.00	5.00(30.46)	62.00(27.50)	33.50	2.99
	Average	0.00	95.71	98.57	97.14	24.00(33.43)	53.00(33.89)	38.43	2.53
*H. brevisubulatum*	1H	0.00	100.00	91.67	95.84	2.00(35.09)	15.00(39.14)	8.50	11.27
	2H	0.00	91.67	83.33	87.50	0.00	25.00(36.37)	12.50	7.00
	3H	0.00	97.22	90.28	93.75	0.00	20.83(42.61)	10.42	9.00
	4H	0.00	100.00	100.00	100.00	0.00	25.00(42.56)	12.50	8.00
	5H	0.00	95.83	100.00	97.92	0.00	16.67(45.05)	8.33	11.75
	6H	0.00	100.00	100.00	100.00	0.00	20.83(38.88)	10.40	9.62
	7H	0.00	97.22	86.11	91.67	0.00	8.33(46.30)	4.17	21.98
	Average	0.00	97.42	93.06	95.24	0.29(5.01)	18.81(41.56)	9.55	9.97

Data show crossover positions per chromosome per cell. Numbers in parentheses indicate physical locations.

**Table 2:** Chiasmata locations and occurrence frequencies (%) at the metaphase I in three species.

Species	Chromosome	Metaphase I							
		No chiamata	Terminal chiasmata			Interstitial chiasmata			Ratio (FT/FI)
			Short-arm	Long-arm	Average	Short-arm	Long-arm	Average	
*H. bogdanii*	1H	0.00	97.00	100.00	98.50	11.00(31.48)	37.00(35.81)	24.00	4.10
	2H	0.00	94.00	100.00	97.00	9.00(34.01)	54.00(36.07)	31.50	3.08
	3H	0.00	97.00	100.00	98.50	14.00(34.24)	69.00(32.82)	41.50	2.37
	4H	0.00	89.00	91.00	90.00	9.00(28.01)	40.00(33.82)	24.50	3.67
	5H	0.00	97.00	91.00	94.00	14.00(30.86)	54.00(38.12)	34.00	2.76
	6H	0.00	97.00	91.00	94.00	0.00	51.00(31.75)	25.50	3.69
	7H	0.00	100.00	97.00	98.50	17.00(34.23)	54.00(31.87)	35.50	2.77
	Average	0.00	95.86	95.71	95.79	10.57(27.55)	51.29(34.32)	30.93	3.10
*E. sibiricus*	1H	0.00	100.00	100.00	100.00	32.00(28.76)	10.00(33.80)	21.00	4.76
	2H	0.00	95.00	95.00	95.00	8.00(25.85)	34.00(28.80)	21.00	4.52
	3H	0.00	92.00	92.00	92.00	8.00(28.21)	47.00(31.37)	27.50	3.35
	4H	0.00	84.00	100.00	92.00	5.00(30.63)	34.00(32.52)	19.50	4.72
	5H	0.00	63.00	100.00	81.50	0.00	66.00(36.86)	33.00	2.47
	6H	42.00	63.00	61.00	62.00	2.00(31.63)	16.00(30.91)	9.00	6.89
	7H	0.00	97.00	95.00	96.00	2.00(25.97)	37.00(26.99)	19.50	4.92
	Average	6.00	84.86	91.86	88.36	8.14(24.44)	34.86(31.61)	21.50	4.11
*H. brevisubulatum*	1H	31.25	84.00	88.00	86.00	0.00	6.00(37.16)	3.00	28.67
	2H	12.50	91.00	97.00	94.00	0.00	14.00(35.30)	7.00	13.43
	3H	6.25	91.00	98.00	94.50	0.00	14.00(40.95)	7.00	13.50
	4H	31.25	97.00	81.00	89.00	0.00	11.00(40.60)	5.50	16.18
	5H	0.00	83.00	97.00	90.00	4.69(30.30)	4.69(44.43)	4.69	19.19
	6H	6.25	84.00	91.00	87.50	0.00	9.00(36.20)	4.50	19.44
	7H	0.00	97.00	95.00	96.00	0.00	20.00(41.01)	10.00	9.60
	Average	12.50	85.00	93.29	89.15	0.43(4.33)	12.14(39.38)	6.29	14.17

Data show crossover positions per chromosome per cell. Numbers in parentheses indicate physical locations.

Statistical analysis of localized chiasmata frequencies revealed that terminal chiasmata decreased from *H. bogdanii* (99.43%) to *E. sibiricus* (97.14%) and *H*. *brevisubulatum* (95.24%), whereas interstitial chiasmata reduced from *H. bogdanii* (52.79%) to *E. sibiricus* (38.43%) and H. *brevisubulatum* (9.55%) across the total H genome chromosomes during the diakinesis. The FT/FI ratio was significantly higher in *H*. *brevisubulatum* (9.97) than in *E. sibiricus* (2.53) and *H. bogdanii* (1.88) (**Table 1**). This indicates that chiasmata frequencies are reduced from diploid to tetraploid species, with autopolyploids exhibiting significantly lower frequencies in both terminal and interstitial regions. Additionally, chiasmata show a shift toward terminal distribution in tetraploids compared to diploids, a trend that is particularly pronounced in the autopolyploid. In metaphase I, the frequency and distribution of chiasmata across the three species were similar to those observed during diakinesis in the total genome (**Table 2**). However, chiasma frequency was lower during metaphase I than during diakinesis in all three species. This reduction can be largely because the partially resolved terminal chiasmata through segregation and the terminalization of interstitial chiasmata. The increasing FT/FI ratio indicated that the chiasmata were more terminally distributed in metaphase I because of chiasmata terminalization. However, the autotetraploid species *H*. *brevisubulatum* exhibited a significant increase.

The frequency of chiasmata occurrence in individual chromosomes generally reduced from diploid to tetraploid, consistent with pattern similar to that observed in the total genome during diakinesis. However, the increases in interstitial chiasmata frequency in the short arm of chromosome 1H—from 34% in *H. bogdanii* to 71% in *E. sibiricus*—and in chromosome 4H—from 34% to 52%—were significantly exceptional (**Table 1**). Additionally, in metaphase I, the frequency of chiasmata occurrence in individual chromosomes generally reduced from diploids to tetraploids (**Table 2**).

A comparison of chiasmata distribution among the homoeologous chromosomes across the three species demonstrated that distal chiasmata were highly conserved, whereas the interstitial chiasmata were variably distributed during diakinesis. At the whole-chromosome level, based on the relative length ratio, the interstitial chiasmata were most proximal in *H. bogdanii* (52.79%), more distal in *E. sibiricus* (38.43%), and most distally in *H. brevisubulatum* (9.55%) (**Table 1**). However, inconsistent patterns of interstitial chiasmata distribution were observed in both the short and long arms. In the long arms, interstitial chiasmata were located more proximally in *H. brevisubulatum* (41.56%) than that in *H. bogdanii* (36.25%) and *E. sibiricus* (33.89%). In the short arms, they were more proximally located in *H. bogdanii* (35.23%) than that in *E. sibiricus* (33.43%), with extreme cases of absence of interstitial chiasmata observed in most chromosomes of *H. brevisubulatum* (**Table 1**). The locations of the chiasmata in metaphase I exhibited patterns similar to those observed in diakinesis but showed a reduced relative length ratio of interstitial chiasmata according to the process of chiasmata terminalization.

## 4 Discussion

Initial polyploids pose significant challenges that often form aberrant associations among the additional homologs (called multivalents) in meiosis I that can cause mis-segregation and reduced fertility (Bomblies et al., 2015). Auto- and allopolyploids may overcome this issue using distinct evolutionary solutions that suppress multivalent associations. In autopolyploids, meiotic stability can be achieved by reducing crossover (Le Comber et al., 2010), primarily by strengthening crossover interference (Morgan et al., 2021). In this study, the global chiasma (cytological crossover) number was significantly reduced from diploid *H. bogdanii* to autopolyploid *H. brevisubulatum* during diakinesis. Specifically, the absence of interstitial chiasmata on the short arms of *H. brevisubulatum* indicated significant crossover interference. However, *H. brevisubulatum* is notably characterized by the prevalence of quadrivalent configurations (44.86%), in contrast to the autopolyploid *Arabidopsis arenosa*, where cytological diploidization predominates and bivalents form the primary pairing configuration (Yant et al., 2013). This indicates that the quadrivalents of large chromosomes in Triticeae species are more challenging to resolve than those from small chromosomes in *Arabidopsis*. In most established allopolyploids, bivalents are formed within each subgenome owing to persistent heterozygosity between distinct subgenomes (Pikaard, 2001; Bomblies and Madlung, 2014). In wheat, genes such as pairing homoeologous 1 (*Ph1*) and *Ph2*, which suppress homoeologous chromosome pairing, play crucial roles in genome stabilization in allopolyploids (Ralph Riley, 1958; Luo et al., 1996; Romero et al., 2001; Serra et al., 2021). Allotetraploid *E. sibiricus* contains distinct H and St subgenomes and exhibits low frequencies of non-homoeologous chromosome exchange in specific cases (Liu et al., 2023). In this study, *E. sibiricus* exhibited disomic inheritance dominated by bivalent formation, suggesting that homeologous chromosome pairing is strictly regulated by genes such as *Ph1* and *Ph2*. Additionally, the number of distinct chiasmata reduced from *H. bogdanii* to *E. sibiricus* across the H genome, indicating that crossover reduction may play a role in the meiotic stability of allopolyploids.

In this study, chiasmata were highly conserved in distal positions but exhibited variability at interstitial positions across the three assessed species during diakinesis. The formation of crossover/chiasmata is influenced globally by multiple factors, including chromatin modification, heterochromatin, and centromere region (Zickler and Kleckner, 2023). During polyploidization, genetic factors can regulate crossover distribution in *trans* (Wang et al., 2021). A comparison of the recombination patterns between wild and domesticated barley landraces revealed high conservation throughout domestication, indicating that the chromatin environment that suppresses recombination is evolutionarily conserved (Dreissig et al., 2019). The variation in chiasmata sites indicates that the chromatin structure of the H genome evolved from diploid to tetraploid species, specifically from diploid to autoploid, and *trans* factors emerging during polyploidization may also play an important role in the chiasmata redistribution in tetraploids. A biased distribution of chiasmata was observed in the three different ploidy species, consistent with crossover-biased localization in distal regions in Triticeae species, such as barley and wheat (Higgins et al., 2014; Osman et al., 2021). Recombination rates vary between and within species (Lawrence et al., 2017; Stapley et al., 2017). Additionally, in this study, the variation in chiasmata occurrence frequency that was used to estimate the variation in recombination was revealed, with a significant reduction from diploid to tetraploid species. Recombination rates are affected by DNA methylation (Habu et al., 2015), histone modifications (Underwood et al., 2018), and nucleosome occupancy (Choi et al., 2017). Global genomic alterations have occurred since the genome shock caused by WGD in polyploids. The genetic and epigenetic roles in the modification of recombination rates during polyploidization require further assessment. Fine-scale estimations of recombination rates in natural populations of wild and domesticated barley have demonstrated that increased recombination rates are shifted toward more distal regions on the long arm of chromosomes in domesticated barley (Dreissig et al., 2019). Differences in recombination rates may have resulted from selection for elevated recombination in genomic regions containing defense response genes during barley domestication (Dreissig et al., 2019). The *H. bogdanii* accession used in this study was collected from a wetland habitat, while the accessions of *H. brevisubulatum* and *E. sibiricus* were obtained from arid grassland environments. Recombination rates of H genome are more distally localized in both *H. brevisubulatum* and *E. sibiricus*, suggesting that the crossover redistribution may be under strong selection and play an important role in species adaptation. Furthermore, significantly increased chiasmata frequencies were detected on the short arms of chromosomes 1H and 4H in *E. sibiricus* compared to *H. bogdanii*. Elucidation of whether this was caused by the internal or external environment will give more clues to the biological significance of crossover redistribution.

Chiasmata terminalization refers to the movement of chiasmata toward the ends of chromatid. As anticipated, chiasmata terminalization was observed from diakinesis to metaphase I in all three species. However, a high frequency (22.61%) of quadrivalent or trivalent configurations was maintained in *H*. *brevisubulatum* at metaphase I, and more pairing configurations were detected in metaphase I than during diakinesis. Frequent chromosomal numerical variations, such as monosomy, nullisomy, and trisomy were identified in *H*. *brevisubulatum* (Dou et al., 2016). This indicates that certain special pairing configurations cannot be completely resolved by chiasmata terminalization. Irregular chiasmata terminalization may lead to homologous chromosome segregation errors and result in aneuploidy.

Polyploid plants, possessing redundant genes, exhibit significant evolutionary and adaptive advantages (Soltis et al., 2015; Heslop-Harrison et al., 2023). Novel genetic variations in polyploid plants can arise from mechanisms such as gene sub functionalization, chromosome rearrangements, gene mutations, and transcriptomic and epigenetic alterations (Liu et al., 2021; Wang et al., 2021; Banouh et al., 2022; Blasio et al., 2022). However, from a macroevolutionary perspective, polyploidy was regarded as an evolutionary dead end (Van de Peer et al., 2017). In meiosis, crossing over is indispensable for the proper segregation of homologous chromosomes and generation of genetic diversity by creating novel gene combinations. In this study, the chiasmata of the H genome were significantly reduced in both number and frequency from diploid to tetraploid. This indicates that although polyploid species can acquire novel genetic variations through WGD, the genetic diversity of their sub genomes may be significantly reduced because of variations in crossover events during polyploidization. Additionally, polyploids are more prone to recurrent deleterious mutations than that of diploids (Otto, 2007; Conover and Wendel, 2022). Deleterious alleles at neighboring loci can hitchhike and reach fixation in regions of low recombination (Hartfield and Otto, 2011), where they tend to be enriched (Zhang et al., 2013). This indicates that the reduction in crossover within sub genomes from diploid to tetraploid species may contribute to the genetic load.

In this study, molecular cytology techniques, such as FISH, were employed to identify each chromosome at different stages of meiosis and observe varied configurations and frequencies. Crossovers or chiasmata can be more accurately detected using immunolocalization of crossover-specific proteins such as MLH1 or HEI10 (Higgins et al., 2014; Osman et al., 2021). A more precise description of variations in chiasmata patterns from diploid to tetraploid can be achieved by combining FISH and immunolocalization techniques.

## Data Availability

The original contributions presented in the study are included in the article/**Supplementary Material**. Further inquiries can be directed to the corresponding author.
