# Supplementary material for: Meiotic chiasmata variations in the H genome among Triticeae species of varying ploidy
Source: Front Plant Sci. 2025 Oct 15;16:1666216. doi: 10.3389/fpls.2025.1666216 (PMC12568566; doi:10.3389/fpls.2025.1666216)
Supplement: Supplementary file 1 [file DataSheet1.docx]

Supplementary Material

# Supplementary Figures and Tables

## Supplementary Figures


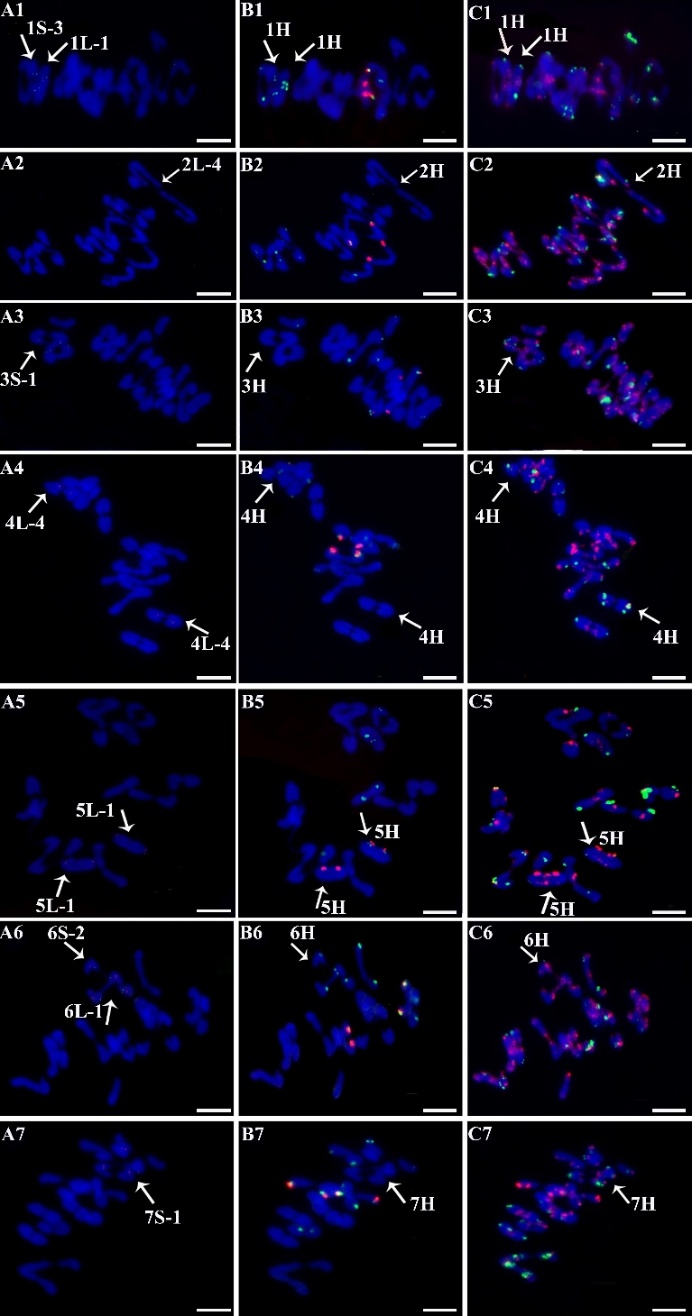


**Supplementary Figure 1.** Patterns of sequential fluorescence in-situ hybridization with single-gene and repetitive sequencies on meiotic metaphase Ⅰ chromosomes of *Hordeum brevisubulatum*.

A1-A7: probed with a single gene; B1-B7: probed with 45S rDNA (red) and 5S rDNA (green); C1-C7: probed with pAs1 (red) and (AAG)_10_ (green). Notes and arrows in A indicate homoeologous chromosomes detected by cDNA probes (Danilova et al., 2014). Notes and arrows in B and C indicate corresponding H genome chromosomes characterized by repetitive sequences probes (Liu et al. 2023) identified chromosomes. Bars = 10 μm.

**
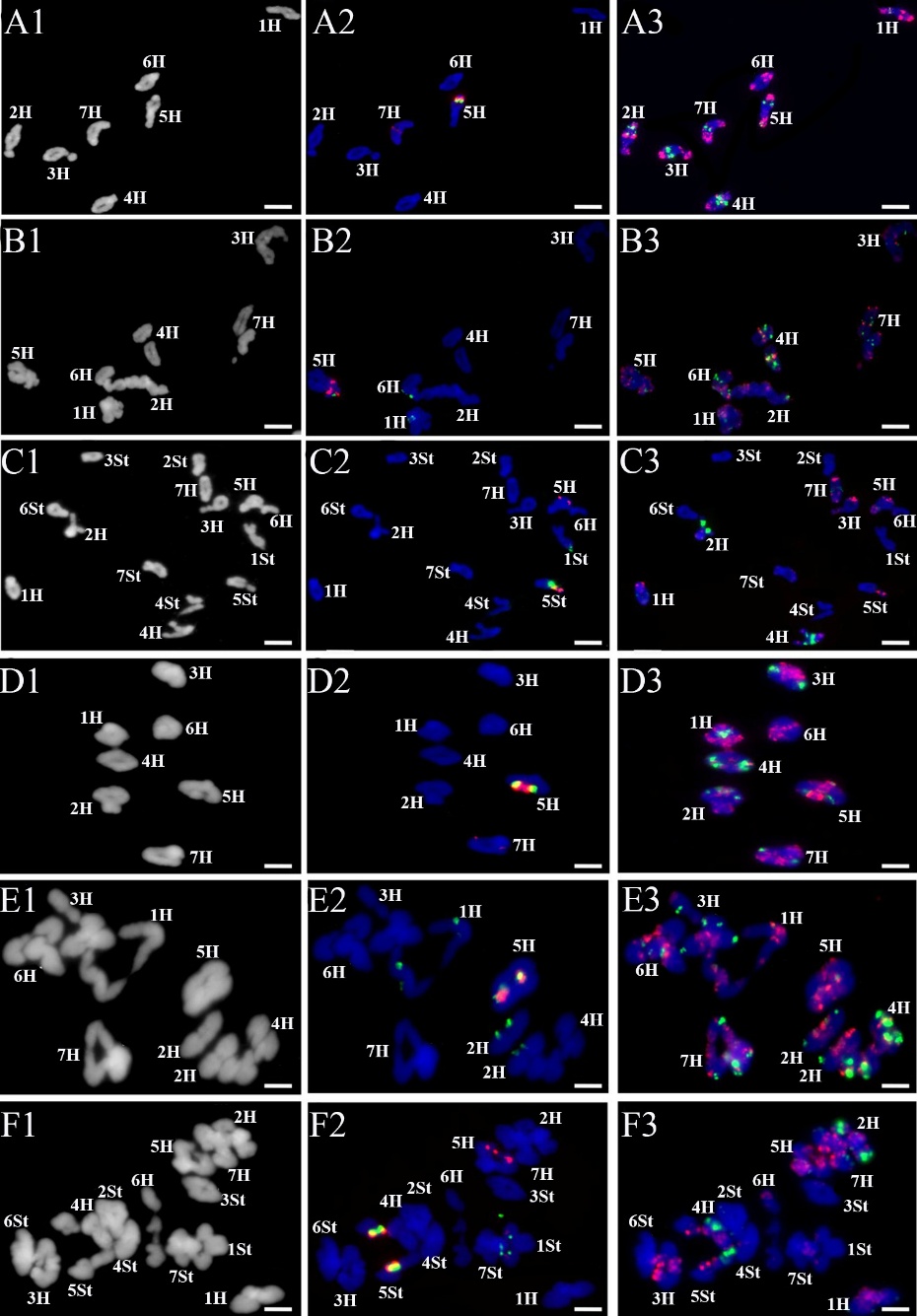
**

**Supplementary Figure 2.** Representative sequential fluorescence *in situ* hybridization patterns on meiotic diakinesis and metaphase I chromosomes of different ploidy species.

A-C: Diakinesis and D-F: Metaphase I. A1-A3 and D1-D3: *H. bogdanii*. B1-B3 and E1-E3: *H. brevisubulatum*. C1-C3 and F1-F3: *E. sibiricus.* Patterns are vertically arranged and stained with 4',6-diamidino-2-phenylindole (A1-F1). Probed with 45S rDNA (red) and 5S rDNA (green) (A2-F2). Probed with pAs1 (red) and (AAG)_10_ (green) (A3-F3). Bars = 10 μm.
